# *Morinda citrifolia* Linn. (Noni) and Its Potential in Obesity-Related Metabolic Dysfunction

**DOI:** 10.3390/nu9060540

**Published:** 2017-05-25

**Authors:** Aline Carla Inada, Priscila Silva Figueiredo, Rosângela Aparecida dos Santos-Eichler, Karine de Cássia Freitas, Priscila Aiko Hiane, Alinne Pereira de Castro, Rita de Cássia Avellaneda Guimarães

**Affiliations:** 1 Post Graduate Program in Health and Development in the Central-West Region of Brazil, Federal University of Mato Grosso do Sul-UFMS, Campo Grande 79079-900, MS, Brazil; inada.aline@gmail.com (A.C.I.); pri.figueiredo92@gmail.com (P.S.F.); kcfreitas@gmail.com (K.d.C.F.); priscila.hiane@ufms.br (P.A.H.); 2 Department of Pharmacology, Biomedical Science Institute, University of São Paulo, São Paulo 05508900, SP, Brazil; reichler@icb.usp.br; 3 Post-Graduate Program in Biotechnology, Catholic University Dom Bosco, Campo Grande 79117-900, MS, Brazil; p.alinne@gmail.com

**Keywords:** *Morinda citrifolia* L., obesity, obesity-related metabolic dysfunction, health

## Abstract

Cultural and economic shifts in the early 19th century led to the rapid development of companies that made good profits from technologically-produced commodities. In this way, some habits changed in society, such as the overconsumption of processed and micronutrient-poor foods and devices that gave rise to a sedentary lifestyle. These factors influenced host-microbiome interactions which, in turn, mediated the etiopathogenesis of “new-era” disorders and diseases, which are closely related, such as obesity, type 2 diabetes mellitus, non-alcoholic fatty liver disease, hypertension, and inflammatory bowel disease, which are characterized by chronic dysregulation of metabolic and immune processes. These pathological conditions require novel and effective therapeutic approaches. *Morinda* *citrifolia* (noni) is well known as a traditional healing plant due to its medicinal properties. Thus, many studies have been conducted to understand its bioactive compounds and their mechanisms of action. However, in obesity and obesity-related metabolic (dysfunction) syndrome, other studies are necessary to better elucidate noni’s mechanisms of action, mainly due to the complexity of the pathophysiology of obesity and its metabolic dysfunction. In this review, we summarize not only the clinical effects, but also important cell signaling pathways in in vivo and in vitro assays of potent bioactive compounds present in the noni plant which have been reported in studies of obesity and obesity-associated metabolic dysfunction.

## 1. Introduction

The prevalence of obese individuals has doubled worldwide since the 1980s. In 2014, it was estimated that more than 1.9 billion adults were overweight, corresponding to 39% of all adults in the world. Of this latter group, 13% were already considered obese, i.e., 600 million [1]. In the United States (USA), obesity is a problem that increased by approximately 50% among adults throughout the 1980s and 1990s [2] and may result in a reduction in longevity of the North American population [3].

Obesity is prevalent in low-income groups who often live in urban areas in the USA and Europe. According to the World Health Organization (WHO), in 2015, more than 50% of adults were overweight or obese in 46 countries across Europe, especially in the eastern part of the region. Nowadays, overweight and obesity are estimated to result in the deaths of about 320,000 men and women in 20 countries in Western Europe every year [4]. In 2012, China’s Minister of Health indicated that in the country with 1.2 billion individuals, 300 million Chinese were obese [5]. In Brazil, more than a half of the Brazilian population was overweight (52.5%), of which 13.9% were considered obese in 2014 [6].

Although obesity is the result of an imbalance between energy intake and expenditure, it is likely that a disturbed metabolism due to inadequate nutrition contributes to an abnormal or excessive fat accumulation associated with impaired health. Central obesity is a sign of the most prevalent chronic metabolic disorder of our era with important global public health challenges. Genetic factors, together with inappropriate food supply, entertainment, and labor-saving devices, and the advertising of highly-appealing foodstuffs by the food industry, results in energy-dense diets and decreased physical activity. This constitutes a gene-environment interaction, where endocrine factors mediate and stimulate some of the pathways that lead to obesity [1,7,8]. Recently, many groups have been focusing their studies on the involvement of the gut microbiota in obesity and metabolic dysfunction. In 2006, one of the first studies that found a relationship between gut microbiota and weight gain reported that the latter was putatively caused by an increase in energy-harvesting capabilities of the microbiota in obese persons [9].

Central, as opposed to peripheral, obesity predisposes individuals to metabolic abnormalities, cardiometabolic complications, such as insulin resistance, type 2 diabetes mellitus (T2DM) dyslipidemia, hypertension, and non-alcoholic fatty liver disease (NAFLD), which are components of obesity-related metabolic syndrome (MetS) that put individuals at risk of developing cardiovascular disease (CVD) [10,11]. Nowadays, the term ‘metabolic syndrome’ is used widely and is defined as when an individual shows at least three of the following cardiovascular risk factors: central obesity (excessive upper body and visceral fat), dyslipidemia (high triglycerides or low high density lipoprotein cholesterol (HDL) levels), or hypertension or hyperglycemia (T2DM) [12].

There are various pharmacological and non-pharmacological options which are broadly accepted as treatment and prevention of obesity and obesity-related diseases, including dietary control, physical activity, lifestyle changes, weight-loss medications, weight-loss surgeries, or specific medications [13]. Various dietary patterns have been extensively studied for health promotion and to diminish the risks of chronic diseases. A “healthy eating concept” has been put forward in which functional foods and nutraceuticals are important parts [14].

In this context, according to Health Canada, the term nutraceutical is conceptualized as a product isolated or purified from foods that is generally sold in medicine formulations not usually associated with food. A nutraceutical has been shown to have a physiological benefit or provide protection against chronic diseases. The term ‘functional food’ is distinguished as a food that is similar in appearance to, or may be, a conventional food, that is consumed as part of a usual diet, and has demonstrated physiological benefits and/or to reduce the risk of chronic disease beyond basic nutritional functions [15].

Due to the complexity of obesity pathogenesis, the majority of the treatments are not related to obesity alone, seeing that it is associated with other metabolic disorders, such as oxidative stress and/or inflammatory alterations that can occur concomitantly in several tissues [11]. Recent studies have focused on the development of innovative therapeutic agents from natural sources as an alternative medicine. Nevertheless, the challenge of natural product studies is to evaluate, in a consistent way, the mechanisms of action and bioactive compounds which could assure the beneficial effects of the products [13,16].

*Morinda citrifolia* L. (noni) is an example of a plant used as a functional food and has been widely studied due to its apparent beneficial effects on human health. It has been investigated as an alternative in anticancer, antibacterial, and antimicrobial therapies, and in the treatment of esophageal reflux and ulcers in animals [17,18,19,20,21]. In humans, there are few studies showing the beneficial effects of noni. Sattar et al. [22] and Siddiqui et al. [23] demonstrated the effective benefits of a topical ointment prepared from noni stem extract against cutaneous leishmaniasis. Palu et al. [24] showed a 25% reduction in lipid peroxidation in the blood of athletes, after an endurance test, taking noni juice (NJ) compared to controls, thereby demonstrating an antioxidant effect. The antioxidant properties of noni juice were also demonstrated by Wang et al. [25] in a study involving 132 heavy cigarette smokers. They reported reduced plasma levels of superoxide anion radicals and lipid hydroperoxide, which are considered biomarkers of degenerative diseases associated with cigarette smoking, in smokers who drank NJ compared to smokers who did not. Moreover, Issell et al. [26] reported less fatigue and maintenance of physical functions in patients with cancer.

Recently, more attention has been directed to the anti-obesity properties of the noni plant in animal models. However, studies are still scarce in humans and more investigations are needed.

Therefore, the aim of this review was to assess specific in vitro and in vivo studies related to the mechanisms of action and bioactive compounds that promote the benefits of *Morinda citrifolia* L. (noni) in the treatment of obesity and obesity-related metabolic dysfunction, such as insulin resistance/T2DM, dyslipidemia, hypertension, NAFLD [27,28], and its influence on the gut microbiota.

## 2. *Morinda citrifolia* Linn. (Noni)

The genus *Morinda* belongs to the Madder or Coffee family Rubiaceae and includes approximately 80 species, including *Morinda citrifolia* Linn (*Morinda citrifolia* L.), which is popularly called noni or Indian Mulberry [29]. Noni was used as a medicinal plant by the Polynesians 200 years ago, but it has never been a traditional food, although it has been called as a starvation fruit. Currently, noni is a typical plant found in tropical climate regions of the USA, such as Hawaii, to Brazil, reaching Tahitian, Malaysia, and Fiji Islands [30,31]. The first idea of potential benefits of noni fruit started with Heinicke [32] who demonstrated that noni contained the alkaloid xeronine. Even though noni fruits showed insignificant amounts of free xeronine, they contained considerable amounts of the precursor of xeronine, which was named proxeronine. One of the explanations for the medicinal action of noni fruits is that xeronine could modulate the conformation and stability of specific proteins. Heinicke described beneficial effects of noni fruits, such as in menstrual cramps, hypertension, burns, depression, atherosclerosis, digestion, relief for pain, and many others.

In 1996, because of the nutraceutical and therapeutic properties of noni, commercial NJ was marketed as a dietary supplement. Afterwards, in 2003, the European Commission approved Tahitian noni juice as a novel food by the Health and Consumer Protection Directorate General. Many investigators have studied the bioactive compounds present in noni fruits, as well as in other parts of the plant, such as leaves, roots, roots bark, seeds, stems, and flowers, because of their potential benefits to health [33,34,35].

On the other hand, noni fruit and juice display some challenging peculiarities, such as a bitter and astringent flavor, as well as a strong rancid odor, which prompts some companies to change these organoleptic properties to create a more palatable product. These companies have been producing flavored NJ with the addition of other fruit juices to create a better-tasting product [36]. Other issues concerning the noni plant are the toxicity, adverse side-effects, the safety of long-term consumption, bioactive compounds, and mechanisms of action, which are important factors to be elucidated in in vitro and in vivo studies to be totally acceptable for human consumption [37].

### 2.1. Nutritional Values of the Noni Plant

Relevant nutritional and chemical analyses have demonstrated that noni fruit contains 90% water and 10% dry matter. The dry matter is composed of soluble solids, dietary fibers, and proteins. Chunhieng et al. [38] reported that 5% of soluble solids are reducing sugars (glucose and fructose) and 1.3% is sucrose. Approximately 11.3% of the dry matter is protein and the main amino acids are glutamic acid, aspartic acid, and isoleucine. Moreover, 10–12% are minerals, which include calcium, sulfur, potassium, magnesium, sodium, phosphorus, and traces of selenium. The main vitamins reported in noni fruit puree are ascorbic acid (vitamin C), which corresponds to 250 mg ascorbic acid per 100 g fresh matter, niacin (vitamin B3), and vitamin A [33,39,40].

### 2.2. Chemical Composition of the Noni Plant

Despite the issues surrounding *Morinda citrifolia* fruit, especially its taste and odor, people still use the bottle-pasteurized juice, either in pure form or mixed with other juices, due to the various phytochemicals in the noni plant totaling approximately 200 compounds. These bioactive compounds are present in different parts of the plant. Noni fruit and other parts of the plant contain large amounts of phytochemicals, including phenolic compounds, anthraquinones, carbohydrates, organic acids, alcohols, vitamins, flavonoids, iridoids, ketones, lignans, triterpenoids, nucleosides, sterols, fatty acids, carotenoids, and many others [29,35,37].

The content of phenols, antioxidants, and ascorbic acid present in the noni fruit increase from the green to white hard stage, whereas they diminish from the white hard stage to the ripe/soft stage [36,41]. In the white hard stage, noni fruits have approximately two times more antioxidant activity, 1.5 times higher phenol content, and seven times higher ascorbic acid content, in comparison to immature green fruits, which have 1.1–1.5 times the antioxidant activity, 1.3 times the total phenols, and 1.3 times the content of ascorbic acid [36,42].

### 2.3. Important Phytochemicals of Morinda citrifolia on Obesity and Obesity-Related Metabolic Dysfunction

The high prevalence of obesity and obese-related metabolic dysfunction has led to extensive investigations to seek out alternative therapies because of several reports of side-effects that are promoted by synthetic drugs. Noni can provide important natural products that have been widely studied and may be considered an alternative therapy for many diseases [14]. Many scientific publications have shown that the noni plant contains a variety of nutritional and functional compounds. However, our current knowledge of these compounds is still not satisfactory. Some studies have demonstrated that the principal bioactive compounds from *Morinda citrifolia* have potentially beneficial effects in obesity and obesity-related metabolic dysfunction, and they are listed in Table 1.

These bioactive compounds, called phytochemicals, include phenolic acids, lignans, flavonoids, flavones, flavans-3-ol, anthocyanins, phytosterols, alkaloids, vitamins, and minerals. In recent decades, polyphenols have been the most important compounds shown to possess beneficial effects against obesity and metabolic dysfunction. Polyphenols include phenolic acids, flavonoids, and stilbenes, which are the most common compounds used in the development of natural products for metabolism-associated disorders/diseases [13,44,45].

Phenolic acids are divided into two classes: hydroxycinnamic and hydroxybenzoic acids. Hydroxycinnamic acids include *o*-coumaric acid, *m*-coumaric acid, caffeic acid, ferulic acid, and sinapic acid, and are present in the form of simple esters with glucose or quinic acid. Hydroxybenzoic acids include salicylic acid, gentisic acid, *p*-hydroxybenzoic acid, gallic acid, vanillic acid, and 3,4-dimethoxybenzoic acid. The most common acid derivative is chlorogenic acid [13,44,45].

Flavonoids are abundant compounds in nature and they are divided into six subgroups: flavonols, flavanones, isoflavanoids, flavones, flavand-3-ol, and anthocyanins. Flavonoids have been widely studied due to their therapeutic properties in the treatment of metabolic disorders due to their ability to modulate the numbers of cell signaling pathways that affect carbohydrate digestion, fat deposition, and the release rate of insulin or glucose uptake in insulin-responsive tissues [44,45,50,51].

Quinones are a class of organic compounds, where 9,10-anthraquinones (9,10 dioxoanthracenes) are an important subgroup [74]. Anthraquinones in *Morinda citrifolia* are found especially in the roots, and the main compounds with important effects on metabolism are alizarin, lucidin 3-*O*-β-d-primeveroside, damnacanthol-3-*O*-β-d-primeveroside, and rubiadin-1-methyl ether [72,73].

Coumarins are found in many edible plants and fruits. One of the most important coumarins found in noni plant is scopoletin (6-methoxy-7-hydroxycoumarin), which has shown a notable effect in the treatment of obesity and metabolic dysfunction [49,55]. Lignans and neolignans are present in many plants, being a large group of natural products derived from the oxidative coupling of two C6-C3 units [75]. The most important lignans isolated from noni fruit are americanin A, americanol A, episesamin 2,6-dicatechol, isoprincepin, lirioresinol, lirioresinol B dimethyl ether, morindolin, and 3,3’-bisdemethypinoresinol. These lignans improve parameters in obesity and associated disorders/diseases [58,59,61,76].

Triterpenoids are the largest class of secondary metabolites produced by plants. Ursolic acid and related triterpene compounds, such as oleanolic acid, betulinic acid, uvaol, and α- and β-amyrin are widespread in many plants [77,78]. The most abundant triterpenoid from noni is ursolic acid, which has been widely investigated because of its hypoglycemic property [61,67,68].

Iridoid is a monoterpene, differing from triterpenes, and it is derived from geraniol. Iridoid glucosides and glycosides, a subclass of iridoid, are terpenes bound to glucose, or any sugar, respectively [79]. Asperulosidic acid is one of the most important iridoids isolated from *Morinda citrifolia*, and it has been shown to improve blood fluidity, which influences the health of obese patients and those with obesity-associated disorders, such as hypertension, diabetes and dyslipidemia [57,80,81]. Vitamins (C, ascorbic acid, and E, α-tocopherol) are two important non-enzymatic antioxidants that have important effects because of their free-radical scavenging property [70,71].

### 2.4. Toxicity of the Noni Plant

The toxicity and low palatability could explain why noni has never been a food plant; therefore, considerable effort is needed to extract or deactivate the toxins. Studies have reported human [41,82] and animal [83] toxicity. In some human cases reported, Millonig et al. [41] and Stadlbauer et al. [82] demonstrated that NJ led to signs of hepatoxicity. The first group observed that a 45-year-old man had elevated transaminases and lactate dehydrogenase. After stopping the ingestion of NJ, transaminase levels normalized. The second human clinical study reported that a 29-year-old man, who had previous toxic hepatitis after small doses of paracetamol, developed sub-acute hepatic failure following consumption of 1.5 L of NJ, while a 69-year-old woman without evidence of previous liver disease experienced an episode of self-limited acute hepatitis following consumption of two liters of NJ.

Recently, Shalan et al. [83] compared the chronic toxicity of NJ and noni leaf aqueous extracts (1 and 2 mg/mL, respectively) to drinking water for six months on the liver and kidneys in female mice. This study observed that none of the doses of noni leaf extracts showed toxic effects; however, mice that consumed noni fruit extract at 2 mg/mL showed toxicity symptoms, such as hypoactivity, excessive grooming, sunken eyes, and hunched posture, with 40% mortality after three months of use. The main cause of death was hepatoxicity with dose-dependent hepatocellular necrosis, though with no effects on kidney.

One possible explanation for noni fruit toxicity can be related to the large amount of anthraquinones. Inoue et al. [84] reported that madder dye, a food coloring extract from the roots of *Rubia tinctorium* L., containing large amounts of two anthraquinones, alizarin, and rubiadin (metabolite of lucidin-3-*O*-primeveroside), showed carcinogenicity in the kidney and liver of six-week-old male F344 rats, where rubiadin had higher carcinogenic potential than did alizarin. However, this study was not applied to noni fruit or leaves. West et al. [85] reported that NJ (Tahitian Noni Juice^®^, Tahitian Noni International, American Fork, UT, USA) (TNJ) was not hepatotoxic and demonstrated that anthraquinones did not possess the same universal biological effect and that it was not reasonable to assume that one category of anthraquinones would have the same exact toxic action as another. Westendorf et al. [86], using high-performance liquid chromatography (HPLC), did not detect genotoxic hydroxyanthraquinones (HAs), such as lucidin and rubiadin in NJ.

The same study demonstrated that the treatment of liver cells in vitro (primary rat hepatocytes and H4IIE rat hepatoma cells) with a common TNJ did not induce genotoxicity. To evaluate the possible genotoxicity of TNJ, they used the V79-HPRT assay and ex vivo hepatocyte UDS assay. V79 cells are Chinese hamster fibroblasts and are currently used as a mammalian cell model to determine the mutagenic effect of natural compounds. In V79 cells treated with TNJ no mutagenicity was observed. Moreover, when primary hepatocytes from rats treated with 10 g/kg body weight, about 700 mL of juice for an adult human, were tested, they showed no acute toxic effects, as seen by UDS (unscheduled DNA synthesis), which is a marker of DNA damage repair [86].

Although there are several controversial studies related to toxicity, some authors suggest a closer evaluation of noni products in general. At the time of NJ production, manufacturers must process the product to remove the toxic principles or deactivate them and test the resulting non-toxic preparations for beneficial activity. Additionally, hygienic measures must be taken to ensure safety for human consumption. Nonetheless, these toxicity studies will be irrelevant if NJ products are obtained using inappropriate processing methods, contain microbial contaminants, or have been adulterated with unsafe ingredients [87].

### 2.5. Therapeutical Use of the Noni Plant

Due to the potential bioactive compounds present in *Morinda citrifolia* fruit that are good for human health, some companies add other fruit juices to provide a flavorful and enhanced product [14,88]. However, not only the fruit, but also other parts of the plant, have phytonutrients with beneficial effects. Growing evidence suggests that noni has important antimicrobial and antibacterial activities. Extracts of noni leaves made with three different solvents, ethyl acetate extract, *n*-butanol, or water, showed a very strong antimicrobial and antibacterial activity against some microorganisms, including *Proteus vulgaris*, *Staphylococcus aureus*, *Bacillus subtilis*, and *Escherichia coli* [17].

It has long been believed that noni leaves have a large number of phenolic compounds, especially coumarins and flavonoids, and also acubin, l-asperulose, alizarin, scopoletin, and other anthraquinones [29,89]. These phenolic compounds possess antimicrobial activity [18], which may, due to their antioxidant effects, possibly involve proton exchange processes [90]. The use of noni for esophageal reflux and gastric ulcerative disease has also produced good results, such as preventing the occurrence of esophagitis due to acid reflux, reducing the formation of acute gastric lesions induced by ethanol, suppressing the development of gastric lesions, and also significantly inhibiting gastric acid secretion and pepsin activity in the pylorus-ligated rat [19]. Scopoletin is the component of noni that is thought to have this valuable potential preventive and therapeutic action for gastro-esophageal inflammation [19].

Palu et al. [91] reported another anti-inflammatory action of NJ, where they demonstrated suppression of IL-4 in mice with NJ treatment in relation to water. NJ increased the production of IFN, which is correlated with the activation of macrophages. The suppressive effects of NJ on IL-4 production in splenocytes, concomitant with increased production of IFN, indicates that NJ modulates the immune system.

A recent study reported by Shalan et al. [92] demonstrated that noni leaf extract possessed ergogenic effects, helping delay fatigue by enhancing energy production, regulation, and efficiency in female mice after a swimming endurance test. Noni extract enhanced performance by improving angiogenesis in skeletal muscle and liver (via vascular endothelial growth factor A, VEGFA), showing antioxidant (superoxide dismutase–SOD2 and glutathione-GSH) and anti-inflammatory (IL-4 and IL-10) properties, and ameliorating mitochondrial biogenesis (via AMP-activated protein kinase (AMPK), uncoupling protein-3 (UCP-3), peroxisome transcriptional proliferator-activated receptor gamma, coactivator 1 alpha (PGC-1α), and nuclear respiratory factor-2 (NRF2)) and stress response (cortisol).

Another application of NJ was related to its anticancer properties since it reduces free radicals which are involved in oxidative damage and lipid peroxidation. One of the main components associated with this property is damnacanthal, a valuable anthraquinone found in the roots of the noni plant, which is widely used for the treatment of chronic diseases, such as cancer and heart disease. Damnacanthal is involved in the K-ras pathway, inducing actin fiber organization, which is affected in activated ras-expressing tumors [20,21]. The noni roots also show antispasmodic, vasodilator, and cardiodepressant activities [93].

A phase one clinical trial was conducted to determine the best dose of noni for capsule supplementation, in which a conventional dose escalation design was used to begin, where the subjects of the research were patients with some type of cancer. No adverse effects were found, and the only hurdle was the number of capsules to be ingested to complete the full dose of 14 g of encapsulated freeze-dried noni fruit per day. In addition, some quality of life factors, such as physical function and fatigue control, were improved in patients who took a mean dose of 8 g per day, compared with patients who took higher and lower doses [26].

## 3. Effects of Bioactive Compounds from the *Morinda citrifolia* L. Plant on Obesity and Obesity-Related Metabolic Dysfunction

### 3.1. Morinda citrifolia and Obesity

Obesity is characterized by the expansion of adipose tissue. This type of tissue can be classified as brown, beige, and white adipose tissue (WAT). WAT is considered not only as an energy reservoir, but also as an organ with endocrine functions. It is classified according to its localization as subcutaneous adipose tissue or visceral adipose tissue, the latter being one of the most important fat deposits associated with metabolic disease [94,95,96,97,98,99]. The endocrine functions that revolve around WAT are due to their capacity to maintain, under physiological conditions, lipid metabolism, such as lipogenesis, lipolysis, and adipogenesis processes [97,98,99], and releasing adipokines, which are substances with important biological and metabolic functions, such as adipsin, tumor necrosis factor-α (TNF-α), leptin, adiponectin, monocyte chemoattractant protein-1 (MCP-1), interleukins (IL-6, IL-10, IL-1β), plasminogen activator-1 (PAI-1), components of the renin-angiotensin-aldosterone system (RAAS), resistin, visfatin, omentin, and many others [100,101,102,103,104,105].

In this regard, natural products, such as plants, herbal supplements, and diet-based therapies have been widely studied because of their potential benefits in human health against obesity and its metabolic disorders [106,107,108,109]. Nishioka et al. [110] investigated the mechanisms underlying the beneficial effects of NJ with focus on glucose and lipid metabolism in high-fat diet (HFD) obese C57BL/6 mice (Table 2). The animals that consumed HFD + NJ showed decreased adipose tissue weights, plasma triglyceride levels, and improved glucose tolerance without toxicity and displayed a lower final body weight, compared to the HFD group. These benefits in the parameters and biomarkers of obesity demonstrated the anti-obesity effects of NJ.

Accordingly, the benefits of NJ in HFD mice have also been reported by others. The reduction of weight gain and improvement of metabolic parameters, such as total cholesterol, low-density lipoprotein-cholesterol, glucose and insulin tolerance, fasting glucose levels, and hepatic insulin resistance has been seen in rats [111], mice [49], and hamsters [43] (Table 2). No liver damage was observed. One explanation for the effectiveness of NJ is the large amount of phenolic acids present in its composition, including gentisic acid, *p*-hydroxybenzoic acid, and chlorogenic acid (Table 2).

A recent study demonstrated positive effects of *Morinda citrifolia* leaves (MLE) as dried plant material that were extracted with 60% ethanol (MLE 60) in HFD obese Sprague-Dawley male rats. They tested two different doses of MLE and compared those groups receiving MLE with the group receiving a synthetic anti-obesity drug (Orlistat 30 mg/kg). The parameters adiposity, fecal fat content and plasma lipids, insulin, and leptin with the higher dose of MLE (500 mg/kg) group were similar as that in the Orlistat group, except the ghrelin levels, which showed better results with the lower dose of MLE (250 mg/kg). Some metabolic pathways, including glucose metabolism and TCA (tricarboxylic acid) cycle, amino acid metabolism, choline metabolism, creatinine metabolism, and gut microbiome, were analyzed using a ^1^H nuclear magnetic resonance (^1^HNMR)-based metabolomics approaches. Both doses of the extract showed improvement in certain metabolic pathways that were impaired by HFD-induced obesity [112] (Table 2).

Under physiological conditions, lipogenesis and lipolysis are the two primary metabolic events in adipose tissue, and they are orchestrated to maintain lipid homeostasis. Non-esterified fatty acids accumulate in WAT and are esterified into triacylglycerol by lipoprotein lipase (LPL). This process of synthesis of esterified fatty acids (FAs) is called lipogenesis. On the other hand, lipolysis is the mobilization or hydrolysis of triglycerides. The availability of FAs and glycerol are necessary for energy storage. Glycerol is an important substrate for hepatic gluconeogenesis and FAs are important energy substrates for peripheral tissues [97,98,99]. Therefore, LPL is an important biomarker in obesity and it has been reported to be consistently augmented in the adipose tissue of obese subjects [50].

The influence of *Morinda citrifolia* fruit (MFE) and leaf (MLE) extracts on LPL activity were evaluated in vitro by two independent research groups [50,51]. Pak-Dek et al. [50] studied MFE and MLE with green tea (GTE) and catechin extracts on the enzymatic activity of LPL. The data demonstrated that all extracts tested inhibited LPL activity substantially after 30 min of incubation. However, the greatest inhibition of LPL activity was seen with 0.2 mg/mL MLE in a dose-dependent manner when compared to MFE, GTE and catechin.

Sahib et al. [51] evaluated MFE, *Momordica charantia* (MCE) and *Centella asiatica* (CAE) extracts in LPL inhibition and the effects of the extracts in proliferation and differentiation of 3T3-L1 preadipocytes (Table 2). The results showed that 1 mg/mL MFE exerted the most significant inhibitory effect on LPL, and in a dose-dependent manner. On the other hand, after 24, 48, and 72 hours of extract incubation, only MCE inhibited adipogenesis in the concentration range of 0–5 mg/mL and differentiation at the highest concentration of 0.5 mg/mL at 48 h. Interestingly, the data revealed that all of the extracts contained high concentrations of phenolic compounds, including catechin and epicatechin, which may be the responsible agents for these effects [51].

Several studies attributed these effects on lipid metabolism to the phenolic compounds, especially catechins present in the extracts [113,114]. However, due to the fact that catechins in MFE and MLE were lower than in GTE [50], one explanation about the inhibition of LPL may be the synergistic effect of catechin with other components present in the extracts since synergism between flavonoids is believed to be better than with one alone. In fact, low-processed whole plant extracts supply multiple chemicals, as much as food does, and depends on synergistic metabolic effects to confer health. In conclusion, the groups suggested that MLE and MFE may be used as anti-obesity agents [50,115].

Polyphenols have been intensively used in studies of obesity and weight management, as well as in other metabolic conditions [13,44,45]. The most used polyphenols include phenolic acids (gentisic acid, *p*-hydroxybenzoic acid, and the derivative chlorogenic acid) and flavonoids (epicatechin, catechin, rutin, quercetin, and kaempferol). Several transcriptional factors, such as proliferator-activated receptor (PPAR)-γ and CCAAT/enhancer-binding proteins (C/EBPs), are involved in the early stage of adipocyte differentiation [116]. PPAR-γ, for instance, influences glucose homeostasis and insulin sensitivity [117].

Flavonoids and phenol acids were able to inhibit adipogenesis in 3T3-L1 adipocytes [45]. The polyphenols rutin (flavonoid) and o-coumaric acid (phenol acid) showed the best results in the inhibition of differentiation with lower levels. Moreover, these compounds were able to inhibit the expression of PPAR-γ and C/EBPα protein levels, demonstrating that these polyphenols inhibit adipogenesis by affecting the transcriptional factor cascade upstream of PPAR-γ expression and also inhibiting the expression of leptin and upregulating adiponectin protein levels [45].

Chlorogenic acid has been claimed to modulate lipid and glucose metabolism in vivo in healthy, as well as in metabolic disorder conditions [46,47]. Eight weeks of treatment with chlorogenic acid exhibited important alterations in a model of HFD-obese male golden hamsters, decreasing body weight gain and visceral adiposity, and ameliorating several metabolic parameters. Furthermore, chlorogenic acid modified lipid and glucose metabolism due to (PPAR)-α action which, in turn, regulated binding, transport, oxidation, and synthesis of free fatty acids (FFAs) [46]. Thus, after activation of PPAR-α, the activity of FFA oxidation enzymes may increase elevating fat energy utilization in the liver and muscle, ameliorating insulin tolerance, and decreasing insulin resistance [118,119].

Additionally, another flavonoid that was isolated from the fruit and leaves of *Morinda citrifolia* is kaempferol [52,66,120]. This flavonoid is the major component of soy leaves (SLE), and a recent study evaluated the anti-obesity effects of SLE extracts in HFD-obese male C57BL/6 mice. Ten weeks of treatment suppressed body weight gain and fat accumulation of WAT. Furthermore, kaempferol supplementation (50 mg/kg/day) induced (i) a decrease in pro-inflammatory cytokine (TNFα and IL-6) gene expression; (ii) a downregulation of adipogenesis-related genes, such as C/EBP-α, sterol regulatory element-binding protein-1 (SREBP-1) and fatty acid synthase (FAS); and (iii) an upregulation of fat oxidation-related genes, such as hormone-sensitive lipase (HSL), carnitine palmitoyl transferase 1 (CPT-1), and uncoupling protein-2 (UCP-2), in WAT from HFD-obese mice. Similar results were observed in 3T3-L1 adipocytes, as well [121].

### 3.2. Morinda citrifolia *L*. and Insulin Resistance/Type 2 Diabetes Mellitus (T2DM)

Obesity can lead to insulin resistance, which is a condition in which a cell, tissue, or organism fails to respond appropriately to a given dose of insulin. To understand the mechanisms of insulin resistance, investigators have developed numerous models of insulin resistance using various chemicals, drugs, and nutritional challenges [122]. T2DM is a difficult problem that has been increasing rapidly, and insulin resistance has an important role in the pathogenesis of T2DM. The reconstitution of insulin sensitivity is an important strategy for the treatment of T2DM. Thus, *Morinda citrifolia* has been widely studied as an alternative treatment for these complications.

Intensive research efforts have evaluated the positive effects of *Morinda citrifolia* on glucose homeostasis in models of T2DM [49,61,73,76]. Nguyen et al. [61] observed that methanolic *Morinda citrifolia* extract (part not identified) showed an anti-diabetic effect in vitro. The extract exhibited stimulatory effects on glucose uptake using a fluorescent-tagged glucose probe (2-NBDG) in 3T3-L1 adipocyte cells. The group identified two new lignans, three new neolignans, and 10 known compounds, where the lignans and ursolic acid were the bioactive compounds that confirmed the inhibitory effects on protein tyrosine phosphatase 1B-gene (PTP1B) and stimulatory effects on 2-NBDG. In this study, lignans, such as episesamin 2,6-dicatechol, lirioresinol B, lirioresinol B dimethyl ether, and ursolic acid, were considered the anti-diabetic effectors for the inhibition of PTP1B (Table 3). Protein tyrosine phosphatases (PTPs) are a group of proteins that participate in intracellular signaling and metabolism by dephosphorylating tyrosine residues. There are several PTPs, where PTP1b has important roles in insulin receptor signaling [123] and is a key regulator of the leptin signaling pathway [124].

In other studies, lignans from *Myristica fragrans* Houtt. (nutmeg) demonstrated strong stimulation AMPK activity in differentiated C2C12 cells. AMPK has been considered as a potential therapeutic target for the treatment of metabolic syndrome, including obesity and T2DM [125]. Ursolic acid is one of the most important triterpenoids isolated from various natural products, including *Morinda citrifolia*. Jayaprakasam et al. [67] isolated ursolic acid, as well as anthocyanins from *Cornus mas* (cornelian cherry), and added them to the HFD for an additional eight weeks. The compounds diminished obesity and glucose intolerance in HFD-obese C57Bl/6 mice to some extent [67]. Indeed, the beneficial effects of acute (three days) and chronic (six weeks) treatment with ursolic acid was also reported by others. These treatments increased skeletal muscle and brown fat metabolism which, in turn, increased energy expenditure. These data were confirmed by the reduction of obesity, glucose intolerance, and fatty liver in HFD-obese C57Bl/6 mice [68].

Another important study demonstrated that fermented noni juice (fNJ) administered to HFD-fed C57Bl/6 male mice reduced body weight and improved glucose and insulin tolerance, as well as fasting blood glucose. These authors detected scopoletin, quercetin, and anthocyanin (cyanidin-3-*O*-rutinoside) in methanolic extracts of fNJ using HPLC. They suggested that the anti-diabetic effects of fNJ may be associated with quercetin and anthocyanin [49] (Table 3). Kampkotter et al. [54,126] demonstrated the properties of quercetin in resistance to oxidative stress in an established model of *Caenorhabditis elegans*, which is an in vivo model that has become increasingly popular to evaluate pharmacologically-active compounds of herbal origin. Quercetin not only had a strong antioxidant capacity, but also prolonged the lifespan of *Caenorhabditis elegans* and was considered a modulator of cell signaling processes to exert its protective properties.

Anthocyanins present in bilberry fruit extract ameliorated hyperglycemia and insulin sensitivity in male KK-Aγ mice, a genetic model of T2DM. Anthocyanins activate AMPK, a signaling pathway important because of its role in the control of hepatic glucose and lipid metabolism [127,128]. These data corroborated with another study [67] that isolated anthocyanins (cyanidin 3-*O*-galactoside, pelargonidin 3-*O*-galactoside, and delphinidin 3-*O*-galactoside) from *Cornus mas* (cornelian cherry). HFD-obese mice that received anthocyanins exhibited a non-obese pattern in the glucose tolerance test while HFD-obese mice showed substantial glucose intolerance [67]. Kaempferol and quercetin isolated from *Euonymus alatus* were shown to improve insulin-stimulated glucose uptake in 3T3-L1 mature adipocytes [53].

In addition, Zhang et al. [62] showed that scopoletin, a phenolic coumarin, had beneficial effects on insulin-resistant HepG2 cells. Insulin resistance was evaluated by measuring PI3K-linked protein kinase B/Akt (Akt/PKB). Thereafter, scopoletin was able to stimulate the reactivation of insulin-mediated Akt/PKB phosphorylation, which was greater compared to the positive control rosiglitazone, a thiazolidinedione and activator of PPARγ that markedly improves insulin and glucose parameters in T2DM patients [129]. In 3T3-L1 adipocytes, scopoletin upregulated the expression of PPARγ2, an isoform of PPARγ that has critical functions in adipocyte differentiation, lipid storage, and glucose metabolism [130].

Accordingly, Lee et al. [76] also reported the beneficial effects of noni fruit in diabetes. They used noni fruit powder fermented by Cheonggukjang, which is a fast-fermented soybean paste, and bacteria, such as *Bacillus subtilis* (KCTC11352BP), *Bacillus sonolensis* (KCTC11354BP), *Bacillus* sp. (KCTC 11351BP) and *Bacillus circulans* (KCTC 11355BP). The data showed that a fNJ (FMC)-based diet, for 90 days was effective in reducing fasting glucose and glycosylated hemoglobin (HBA1c), enhancing insulin sensitivity and decreasing LDL, triglycerides and cholesterol in KK-Aγ diabetic mice. These responses are believed to be due to the activation of PPAR-γ and AMPK phosphorylation (Table 3).

In fact, when HEK293 cells were transfected with a plasmid containing the PPAR-γ response-element-driven luciferase reporter gene, fermented noni extract activated the PPAR-γ-dependent luciferase activity. In addition, this compound stimulated glucose uptake in C2C12 culture cells via activation of the AMPK pathway. These effects could be due to the presence of anthraquinones, flavonoids and terpenoids [76].

The evaluation of dried roots of *Morinda citrifolia* were extracted with methanol, suspended in water (H_2_O), and partitioned in different parts of chloroform (CHCl_3_), ethyl acetate (EtOAc), and n-butanol (*n*-BuOH). Sequentially, the solvents were removed from these different parts in order to generate the soluble phases: CHCl_3_, EtOAc, *n*-BuOH, and H_2_O. Therefore, the fractions of soluble phases of methanol extract from *Morinda citrifolia* roots (MRE) were administrated orally to streptozotocin-induced ddY diabetic male mice (single administration). Only *n*-BuOH exhibited a significant reduction of blood glucose levels after five hours of administration, whereas methanol extract and other soluble phases did not display any hypoglycemic effects. Hence, after isolation of compounds from the *n*-BuOH fraction, two iridoids and three anthraquinones were identified, where two anthraquinones, lucidin (lucidin 3-*O*-β-d-primeveroside), and damnacanthol-3-*O*-β-d-primeveroside, were responsible for the hypoglycemic effects [73] (Table 3).

Likewise, anthraquinones are important agents in the treatment of diabetes [72]. Three anthraquinones (1,2-dimethoxyanthraquinone, alizarin-2-methyl ether and rubiadin-1-methyl ether) were isolated from the *n*-hexane and CHCl_3_ fractions of *Morinda officinalis* roots and used to investigate fat accumulation in 3T3-L1 pre-adipocytes using the oil red O staining method. Alizarin-2-methyl ether was the compound that produced the highest increase in adipocyte differentiation followed by rubiadin-1-methyl ether and 1,2-dimethoxyanthraquinone [72].

*Morinda citrifolia* also displayed positive effects in streptozotocin (STPZ)-diabetic rats [70,71,131,132]. In diabetic patients, wounds are very complex to manage due to impaired wound-healing. Nayak et al. [131] evaluated the wound-healing effects of NJ on an excision wound model in induced diabetic rats. These animals exhibited improvement in wound-healing after consuming NJ. The wound area was reduced earlier and had less dead tissue at the wound site in NJ-treated rats than in their respective controls. The authors correlated wound-healing improvement with low fasting glucose, which was also found to be reduced. Triterpenoids and tannins are bioactive compounds that promote wound-healing due to their astringent and antimicrobial properties, promoting wound contraction and increasing the rate of epithelialization. Furthermore, these substances, especially triterpenoids, may have hypoglycemic effects [133].

The anti-diabetic effects of fNJ could be seen in another STPZ-diabetic rat model [132]. A possible explanation for these effects was the presence of saponins, triterpenes, steroids, flavonoids (rutin), and cardiac glycosides in the extract. However, the group attributed these effects to triterpenes and saponins, the principal compounds that show the highest specific actions on glucose metabolism. Norberg et al. [69] reported that saponins may have a glucagon decreasing effect and may enhance glucose utilization, thereby lowering blood glucose. Moreover, saponins stimulate insulin release from the pancreas due to diminishing degradation of glucagon-like peptide (GLP). Triterpenoids have already been indicated as beneficial agents in diabetes mellitus, especially in alloxan-induced mice, improving symptoms of glycosuria and elevated blood sugar [134,135].

These data corroborate another study [70] in which aqueous and methanol MFE were administered to STPZ-diabetic rats for one week before diabetes induction, three days during induction, and five weeks afterwards. Both MFE reduced blood glucose, glycosylated hemoglobin, blood urea, and creatinine levels, which were explained by the possible prevalence of antioxidants (vitamin C, vitamin E, flavonoids, terpenoids, and anthraquinones) in these extracts. In the same animal model, an antihyperglycemic effect and antioxidant activity were observed for ethanolic MFE given for 30 days [71].

These antioxidant properties were demonstrated by thiobarbituric acid reactive substance (TBARS), hydroperoxidose, and enzymatic and non-enzymatic antioxidants, such as catalase (CAT), glutathione, superoxide dismutase (SOD), and vitamins C and E, respectively. It is believed that these beneficial effects of noni were due to the synergistic effect of several biologically-active ingredients in the extract, which provides for the antioxidant nature of the extract [71]. Another synergistic effect of components of NJ was observed in alloxan-diabetic Sprague-Dawley rats, whereas NJ given for four weeks combined with insulin was more effective in lowering fasting glucose levels compared to the use of NJ or insulin alone [136].

### 3.3. Morinda citrifolia and Non-Alcoholic Fatty Liver Disease (NAFLD)

The liver is an important organ that possesses a fundamental role in metabolic homeostasis, such as in the process of lipogenesis, gluconeogenesis, and cholesterol metabolism. In recent decades, a variety of pathological conditions emphasize the importance of metabolic functions that occur in the liver. The increased prevalence of obesity and metabolic syndrome lead to pathophysiological changes that may result in the development of non-alcoholic fatty liver disease (NAFLD) [137].

NAFLD is considered one of the modern diseases of the new era, being the major cause of mortality and morbimortality related to chronic liver diseases. Most of the time, this pathology occurs in 25% of the population, increasing to 70% in obese and T2DM patients [138,139,140]. NAFLD is a liver disease that may progress from hepatic steatosis alone, without inflammation and hepatocellular damage, to steatohepatitis with lobular inflammation, and with evidence of hepatocyte injury called non-alcoholic steatohepatitis (NASH). Many patients that have NASH develop liver fibrosis, which may result in hepatocyte death, cirrhosis, and hepatocellular carcinoma, with high chances for the need of liver transplantation [141].

Despite some studies having demonstrated hepatotoxic effects of noni in humans and animals [41,82,83], a few others have reported hepatoprotective effects of noni, but it has only been explored in obese animals [43,51]. The effects of *Morinda citrifolia* in NAFLD was performed by Lin et al. [43]. They reported that HFD-induced obese male Golden Syrian hamsters supplemented with different doses of NJ showed diminished biomarkers of liver damage, namely alanine transaminase (ALT), along with diminished TNF-α, IL-1β, inducible nitric oxide synthase (iNOS), cyclooxygenase 2 (COX-2), and metalloproteinase 9 (MMP9) levels, and improved morphological characteristics of hepatic steatosis, such as a decrease in microvesicular steatosis and blurred cellular boundaries. In addition, NJ supplementation in HFD-obese hamsters decreased serum and liver total cholesterol and triglycerides, improved liver antioxidative capacity (CAT, SOD, glutathione peroxidase (GSH-Px), GSH, trolox equivalent anti-oxidative capacity (TEAC)) and lowered liver lipid peroxidation (TBARS) (Table 4).

All of these beneficial effects are possibly due to the large amount of phenolic acids present in NJ. Large amounts of phenolic acids, such as gentisic acid, p-hydroxybenzoic acid, and chlorogenic acid, are the dominant compounds in this juice [43]. Many studies have demonstrated that phenolic compounds act as reactive oxygen species (ROS) scavengers, reducing lipid peroxidation, as well. In vitro studies conducted by Joshi et al. [142] pointed to gentisic acid as the antioxidative and ROS scavenging agent. If those effects are also beneficial to humans it remains unknown and further study is necessary.

The effects of noni juice compounds have been extensively studied in animals. Chlorogenic acid was able to enhance the activity of the important antioxidant enzymes SOD, CAT and GSH-Px in STPZ-nicotinamide-induced type 2 diabetic rats [143]. Complementary studies reported that aqueous extract of *Mesona procumbens*, which has chlorogenic acid as a major compound, has anti-inflammatory action via the upregulation of antioxidants and downregulation of pro-inflammatory biomarkers (TNF-α, iNOS and COX-2) [144], and it exhibits anti-obesity effects and improves lipid metabolism [145].

In corroboration of the hepatic benefits of fNJ supplementation in an obese model, Nerurkar et al. [49] demonstrated that fNJ produced positive effects on plasma glucose levels by modulating hepatic gene expression of phosphoenolpyruvate carboxykinase (PEPCK), glucose-6-phosphatase (G6P) and glucokinase (GCK). PEPCK and G6P are important gluconeogenic enzymes regulated by insulin. They were inhibited after fNJ supplementation, which was confirmed with HepG2 culture cells treated with FOXO1 siRNA and fNJ. GCK was upregulated by fNJ via forkhead box O1 (FOXO1) transcription factor phosphorylation. The hypoglycemic properties of fNJ were associated with the inhibition of hepatic FOXO1 mRNA with concomitant increase in FOXO1 phosphorylation. Consequently, fNJ improved hepatic insulin resistance indicated by homeostatic model assessment-insulin resistance (HOMA-IR) (Table 4).

Those effects may be attributed to flavonoids, quercetin, and anthocyanins, specifically cyanidin-3-*O*-rutinoside, which were isolated from methanolic extracts of fNJ. Some studies demonstrated the inhibitory effect of anthocyanins on oxidative stress via FOXO transcription factor regulation in *Caenorhabiditis elegans* [54,126]. In support of these studies, fNJ promoted the reduction of hepatocyte fatty degeneration (smaller fatty globules and less numerous) in a model of STPZ-diabetic rats. It was suggested that the hepatoprotective activity of *Morinda citrifolia* was due to the antioxidant activity of flavonoid constituents [132].

Anthocyanins (delphinidin and cyanidin) isolated from *Hibiscus sabdafera* extract (HSE) also showed positive effects against obesity and liver damage in HFD-obese hamsters. HSE and anthocyanins regulated total body weight and visceral fat, reduced serum cholesterol and triglyceride levels, protected against oxidation-associated damage in liver by regulating a liver antioxidant enzyme (paraoxonase 1), and also reduced liver damage biomarkers ALT and AST [146].

Another study evaluated the benefits of scopoletin in reducing obesity and liver damage by supplementing the diet with two doses of scopoletin in a HFD model of obese mice. Supplementation resulted in reduced body weight, visceral fat, pro-inflammatory adipokine serum levels (leptin, MCP-1, TNF-α, IL-6, IFNγ), insulin resistance, and hepatic lipid accumulation and, on the other hand, increased serum adiponectin and fecal lipid levels. Moreover, supplementation was able to downregulate genes, such as CIDEA (cell death-inducing DFFA-like effector A) and Apoa4 (apolipoprotein A-IV), which are known to be related to hepatic steatosis and inflammation [147].

### 3.4. Morinda citrifolia and Dyslipidemia/Hypertension

Atherosclerosis is the primary cause of heart disease and stroke. This problem is most common in obese, hypertensive, dyslipidemic, and diabetic patients leading to vascular damage [148]. Although hypertension and dyslipidemia are independent risk factors that lead to atherosclerosis, the latter is also a risk factor for CVDs, such as stroke and myocardial infarction and hypertension. In this way, both dyslipidemia and hypertension are important risk factors for the progression and development of atherosclerosis [149,150,151]. Moreover, these factors are serious pathological conditions for endothelium damage, causing cell proliferation, vascular remodeling, apoptosis, and enhancement of cellular permeability with adhesion molecules that bind monocytes and T lymphocytes. The latter cells are redirected into the intima vasculature by pro-inflammatory and chemoattractant cytokines. Hence, monocytes differentiate into macrophages which overloaded excessive oxidized LDL, become foam cells, elaborate cytokines, and then form atherosclerotic plaques [148].

The atherogenic dyslipidemic phenotype is characterized by high plasma triglycerides, low levels of high-density lipoprotein cholesterol (HDL), and excessive LDL. Additionally, postprandial (non-fasting) triglycerides (postprandial hyperlipidemia) are also an important component of atherosclerosis [152]. The modern synthetic drugs that have been used as treatment for lipid abnormalities are effective at reversing the measured signs, such as decreased LDL levels, but are difficult to afford for many patients and are associated with several side-effects [153]. *Morinda citrifolia* has been demonstrated to be an alternative therapy for this problem. A current study evaluated the effects of NJ on serum lipid profiles in 132 heavy smokers (drinking 29.5 mL to 188 mL of NJ per day). Heavy smoker volunteers who drank NJ displayed a reduction in cholesterol levels, triglycerides, and high-sensitivity C-reactive protein (hs-CRP), a decrease in LDL and homocysteine, and an increase in HDL fraction [25].

However, the few human studies available did not address this issue. Thus, clinical trials are necessary to validate the beneficial qualities of *Morinda citrifolia* bioactive compounds in human metabolic diseases.

In animals, a recent study performed by Shoeb et al. [111] demonstrated that supplementation with two doses of fNJ in cholesterol-rich HFD-induced hyperlipidemia rats showed a significant decrease in total cholesterol, triglycerides, and LDL at both doses when compared to the hyperlipidemic group. The decrease in total cholesterol was observed with the lower dose of fNJ reaching similar values as the positive control atorvastatin (10 mg/kg). Furthermore, the lower dose reduced body weight compared to the hyperlipidemic group and it was also comparable to the hyperlipidemic group receiving atorvastatin (Table 5).

These data corroborate the hypolipidemic effects of noni in a work done by Mandukhail et al. [154]. These authors compared ethanolic extracts of different parts of *Morinda citrifolia* and evaluated different doses of MFE, MLE, and MRE extracts in tyloxapol (Triton WR 1339)-induced hyperlipidemia and HFD-induced dyslipidemia models, both in rats. The highest dose of all extracts produced a significant reduction in total cholesterol and triglyceride levels in the WR 1339 rat group. In contrast, the HFD-induced dyslipidemia group showed different results depending on the extracts at the highest dose of each. Both MFE and MLE prevented the rise in total cholesterol, LDL, total cholesterol/HDL ratio, and atherogenic index without significant effects on HDL. However, MLE prevented the increase in glucose levels and body weight in these animals, while MRE was the best extract in this study, which proved to prevent the rise in all lipid and glucose levels and, at the same time, increasing HDL and preventing weight gain, suggesting antidyslipidemic mechanisms for various parts of the noni plant (Table 5).

Hypolipidemic and other positive effects of *Morinda citrifolia* were suggested in two studies [111,154] that demonstrated the presence of strong antioxidant activity in the noni plant. According to previous studies, Kamiya et al. [60] demonstrated the effects of fruits of *Morinda citrifolia* in preventing atherosclerosis. MFE and its soluble phases (CHCl_3_, EtOAc, n-BuOH, H_2_O) inhibited copper-induced LDL oxidation according to the TBARS method. Lignans were isolated in the EtOAc-soluble phase, including 3,3′-bisdemethypinoresinol, americanol A, morindolin, and isoprincepin, which showed remarkable or strong antioxidant activity. Thus, lignan compounds of noni fruit are involved in the prevention of atherosclerosis, likely due to their numerous phenolic hydroxyl groups (Table 5).

Furthermore, Lin et al. [48] evaluated supplementation with fNJ at different concentrations in HFD-cholesterol hamsters. They found that the group supplemented with fNJ displayed hypolipidemic and antioxidative effects, demonstrated by decreases in serum triglyceride, total cholesterol, atherogenic index, malondialdehyde levels, and hepatic lipids, while antioxidant activity (TEAC and GSH) and fecal lipids were increased (Table 5).

To evaluate the effect of fNJ on lipid metabolism and mechanisms of actions, important genes related to lipid homeostasis were evaluated in the liver, which is the major organ in the regulation of lipid homeostasis. The data showed that SREBP-1c was upregulated after fNJ treatment; this is an important transcription factor that stimulates the expression of lipogenic genes, such as that of fatty acid synthase (FAS). This enzyme is responsible for the biosynthesis of FA. In energy expenditure, peroxisome proliferator-activated receptor-alpha (PPAR-α) upregulates uncoupling protein 2 (UCP2), which increases thermogenesis, while reducing the efficiency of ATP synthesis. In this regard, the gene expression of PPAR-α, as well as UCP-2, was upregulated in the liver after fNJ supplementation. The bioactive compounds responsible for the effects of fNJ were gentisic acid, which was the phenolic in the highest amount, followed by *p*-hydroxybenzoic acid and chlorogenic acid [48].

Many alternatives of anti-hypertensive therapies have been widely studied. Accordingly, some authors have focused on *Morinda citrifolia* as an alternative therapy for hypertension. Using an animal model of hypertension, Wigati et al. [55] investigated the action of ethanolic MLE, MFE, and the combination of both on blood pressure in dexamethasone-induced hypertensive rats, where this model is characterized by nitric oxide deficiency and oxidative stress. All extracts decreased blood pressure, but were not able to repair or inhibit renal damage caused by dexamethasone induction. However, the combination of the two extracts had the highest hypotensive activity. According to the study, the phenolic compounds, such as rutin as a marker in MLE and scopoletin in MFE, were the agents responsible for the hypotensive effects (Table 6).

Previous studies have demonstrated that rutin and scopoletin are important phenolic compounds that affect the cardiovascular system, including blood pressure regulation. Rutin possesses renal-protective activity probably by inhibiting ROS production and through antioxidant activities, reducing elevated malondialdehyde levels and restoring depleted manganese-superoxide dismutase (MnSOD) and GSH, with positive effects on biochemical parameters, as well as on the histopathological morphology of the kidneys [56]. Scopoletin also demonstrated hypotensive effects and relaxation of rat aorta. In addition, a possible inhibitory activity of angiotensin converting enzyme-1 (ACE1) was suggested as a property of this phenolic compound [155,156]. Asperulosidic acid, an iridoid glycoside present in MFE, showed substantial positive effects on blood fluidity and improved certain lifestyle-related diseases, such as hypertension, dyslipidemia, and diabetes [80].

MRE showed antispasmodic and vasodilator activities mediated through blockade of voltage-dependent calcium channels in isolated tissues of rats, guinea pigs, and rabbits [93] (Table 6). These effects were mediated by alkaloids, phenolic compounds, sterols, flavonoids, tannins, coumarins, and anthraquinones, which corroborate a study on *Zingiber officinale* Roscoe (ginger) that evaluated its hypotensive effects [157]. The same bioactive compounds that were found in the noni plant were also detected in ginger and could affect isolated tissues of rats, rabbits, and guinea pigs. The crude extract of ginger decreased blood pressure and exhibited a cardiodepressant activity, with the activity being mediated by Ca^+2^ channel-blocking, which was demonstrated when crude ginger extract shifted the Ca^+2^ dose-response curve to the right, mimicking the effect of the positive control verapamil.

Considering that diuretics are used in the treatment of hypertension, Shenoy et al. [63] evaluated the diuretic potential of NJ in normal rats. The effects observed were an increase in urine volume in a dose-dependent manner with augmentation of the diuretic index accompanied by a significant decrease in sodium and potassium ion excretion. The authors demonstrated that the noni plant had aquaretic, instead of diuretic, actions (Table 6). Some authors believe that herbs act only as aquaretic agents, which increase water excretion without affecting renal handling of electrolytes. In other words, aquaretics only increase urine output, acting on the glomerulus, unlike conventional diuretic drugs that act further along the nephron [63,64].

Herbs often contain large amounts of minerals (electrolytes) and noni fruit has a high content of potassium. Hook et al. [65] evaluated the diuretic effect of *Taraxacum officinale* Weber (dandelion) in normal mice and did not observe any significant variation in electrolytes (Na^+^, K^+^, Ca^+2^), but the final volume of urine produced after five hours was greater than with the positive control, furosemide. That study concluded that the high potassium content of dandelion was responsible for any diuretic activity, where dandelion was similar in action to noni fruit and, thus, the increased urinary volume could be suggestive of an osmotic effect [63,65].

### 3.5. Morinda citrifolia and the Effect on Gut Microbiota

There is a vast community of gut microbes [158]. Much has been invested in the search for nutrients that are selective for a favorable modification of intestinal microbiota, especially those able to increase the amount of *Bifidobacterium* and *Lactobacillus*. The dysbiosis of the gut microbiota has been associated with the development and progression of many human diseases. The ratio of some microbiota species are greater in obese, rather than in lean, individuals [159,160]. Noni, and its juice, exhibit antimicrobial properties and high antioxidant activity, which would be beneficial for a healthy intestinal microbiota [161,162].

As observed in Table 7, fNJ showed a probiotic character by allowing a greater growth of *Lactobacillus*, as well as *Bifidobacterium*, species. This is possible because NJ (as a raw substrate) has a fermentative process with lactic acid bacteria (*Lactobacillus casei* and *Lactobacillus plantarum*) or *Bifidobacterium* (*Bifidobacterium longum*) [161,162].

In addition, noni powder also has a prebiotic action. One of the reasons may be the high amount of polysaccharides in the fruit, since carbohydrates, except for starch, act as dietary fiber, coming intact with the gut and contacting the bacterial community present in the intestinal microbiota [164]. The high molecular weight fraction of NJ is mostly composed of pectic polysaccharides, including rhamnogalacturonan, homogalacturonan, and the neutral side chains of (arabino) galactan and arabinan [165].

Another reason may be the high phenolic composition of the fruit, which may also have a prebiotic function [162], such as quercetin and proanthocyanidin [166,167]. Previous studies have also indicated that phenolic compounds can inhibit the growth of pathogens such as *Escherichia coli* and *Helicobacter pylori* [166].

In addition to the microbiota, the size and height of the villi are important for intestinal function. Diet plays an important role in intestinal morphology [163]. The mucus layer in the small intestine protects the epithelial cells of the small intestine and mediates nutrient transport between the lumen and the membrane of the brush border. The ontogeny of the whole gut has extensive implications for intestinal function [168].

Supplementation with 1% noni fruit powder caused an increase in villus height, villus surface area, and crypt depth when compared with control [163]. Although few studies have evaluated noni fruit with regard to the microbiota and intestinal function, the in vitro and in vivo results presented in Table 7 show that noni fruit shows prebiotic activity, when administered alone, and probiotic activity, when used in fNJ, improving bacterial colonization and intestinal morphology.

## 4. Conclusions

Bioactive compounds from natural resources are a promising field of study for alternative medicines, where *Morinda citrifolia* Linn. (noni) is one of these important options. Noni has demonstrated positive effects in metabolic dysfunction, including the regulation of body weight and fat deposits, lipid and glucose metabolism and blood pressure, hepatoprotective effects, and improvement in bacterial colonization of the gut and intestinal morphology. However, *Morinda citrifolia* has never been an important food plant, probably due to its poor palatability and toxicity, in its natural homeland. This review reports the influence of the noni plant and its bioactive compounds, emphasizing the potential mechanisms of action and effects on cell signaling pathways involved in obesity and obesity-related metabolic dysfunction. The noni plant may be processed for some possible medicinal properties after the management of toxicity, or some parts of the plant may be mixed with less nutritional, but appetizing, food for health benefits. Therefore, doses and the time of treatment or long-term supplementation, and also new alternatives in the near future to improve the bioavailability of the product, are very important issues to be evaluated. Finally, since *Morinda citrifolia* contains important bioactive compounds for health, it may be an alternative therapeutic resource with great potential in the treatment of obesity and obesity-related metabolic dysfunction.

## Figures and Tables

**Table 1:** Principal phytochemicals from *Morinda citrifolia* as bioactive compounds against obesity and obesity-related metabolic dysfunction.

Part of Plant	Structural Class	Bioactive Compounds	Concentrations of Bioactive Compounds	References
Fruit	Phenolic acid	Chlorogenic acid	10.49 mg/100 mL [43]	[43,44,45,46,47,48]
		Gentisic acid	19.16 mg/100 mL [43]	[43,44,45,48]
		P-hydroxybenzoic acid	14.12 mg/100 mL [43]	[43,44,45,48]
	Flavonoids	Anthocyanin (cyanidin-3-*O*-rutinoside)	Data not shown	[49]
		Catechin	53.68 mg/g [50]	[50,51]
		Epicatechin	6.8 mg/g [51]	[51]
		Kaempferol	6.4 mg/g [51]	[52,53]
		Rutin	8.06 mg/g [50]	[45,54,55,56]
		Quercetin	7.4 mg/g [51]	[49,54]
	Iridoids	Asperulosidic acid	Data not shown	[48,57]
	Lignans	Americanin A	17.4 mg [58]	[58,59]
		Americanol A	21 mg 60]	[60]
		Isoprincepin	14 mg [60]	[60]
		Lirioresinol B	Data not shown	[61]
		Lirioresinol B dimethyl ether	Data not shown	[61]
		Morindolin	10 mg [60]	[60]
		3,3’-Bisdemethypinoresinol	69 mg [60]	[60]
	Coumarins	Scopoletin	46.1 mg [58]	[49,55,58,62]
	Minerals	Potassium	3900 mg/L [38]	[38,63,64,65]
	Triterpenoids/terpenes	Ursolic acid	Data not shown	[61,66,67,68]
		Saponin	Data not shown	[69]
	Vitamins	Vitamin C	Data not shown	[70,71]
		Vitamin E	Data not shown	[70,71]
Leaf	Flavonoids	Catechin	63.46 mg/g [50]	[50]
		Epicatechin	23.08 mg/g [50]	[50]
		Rutin	6.83 mg/g [50]	[45,50,55]
		Kaempferol	21–80 mg [52]	[52,66]
	Triterpenoids/terpenes	Ursolic acid	Data not shown	[61,68]
Root	Anthraquinones	1,2-Dimethoxyanthraquinone	3.5 mg [72]	[72]
		Alizarin-2-methyl ether	11.3 mg [72]	[72]
		Rubiadin-1-methyl ether	15.5 mg [72]	[72]
		Lucidin 3-*O*-beta-d-primeveroside	Data not shown	[73]
		Damnacanthol-3-*O*-beta-d-primeveroside	Data not shown	[73]
		Morindone-6-*O*-beta-d-primeveroside	Data not shown	[73]
	Iridoid	Asperulosidic acid	Data not shown	[73]
		Deacetylasperulosidic acid	Data not shown	[73]

Principal bioactive compounds from fruits, leaves, and roots of *Morinda citrifolia* used for obesity and obesity-related metabolic dysfunction.

**Table 2:** The effects of administration of the *Morinda citrifolia* L. plant on obesity.

Host	Part of Plant	Dose/Time	Effects	Reference
Mice	Fruit Noni Juice	1.5 µL/g body weight (twice daily)/5 weeks	−Reduced body weight by 40% in mice fed control, while reduced body weight by 25% in HFD mice.	Nishioka et al. [110]
			−Reduced adipose tissue weights, plasma triglycerides and improved glucose tolerance.	
Rats	Fruit Noni Juice	50 mg/kg/day/30 days	−Reduced body weight (better at 50 mg/kg/day dose).	Shoeb et al. [111]
			−Reduced serum total cholesterol, triglycerides and lipid fractions: LDL and VLDL (all doses).	
		100 mg/kg/day/30 days	−Increased lipid fraction HDL (all doses).	
Mice	Fruit Fermented Noni Juice	1.5 µL/g body weight/twice daily/12 weeks	−Inhibited weight gain after 12 weeks.	Nerurkar et al. [49]
			−Improved glucose and insulin tolerance and fasting glucose in HFD-fed C57Bl/6 mice.	
			−Improved hepatic insulin resistance by FOXO-1 and inhibition of PEPCK and G6P (gluconeogenic enzymes).	
Hamster	Fruit Noni Juice	−3 mL (containing 64.23 mg crude polysaccharides/kg body weight/6 weeks.	−Decreased visceral fat in HFD-hamsters (all doses).	Lin et al. [43]
			−Decreased serum and liver lipids: total cholesterol and triglycerides in HFD hamsters (all doses).	
		−6 mL (containing 128.46 mg crude polysaccharides/kg body weight/6 weeks.	−Beneficial effects on liver and hepatic enzymes (ALT) in HFD hamsters (all doses).	
			−Increased antioxidant capacity in the liver in HFD hamsters (all doses).	
		−9 mL (containing 192.69 mg crude polysaccharides/kg body weight/6 weeks)	−Decreased inflammatory biomarkers in the liver (TNF-α, MCP-1, IL-1β) in HFD hamsters (all doses).	
			−Decreased gelatinolytic levels of MMP9 in HFD hamsters (all doses).	
Rats	Ethanolic Extract of Leaves	−250 mg/mL/9 weeks.	−Prevented weight gain, especially MLE 60 500 mg/kg.	Jambocus et al. [112]
			−Positive effects on adiposity, fecal fat content, plasm lipids, insulin and leptin levels, especially MLE 60 500 mg/kg.	
		−500 mg/mL/9 weeks.	−Improved ghreline levels, especially MLE 250 mg/kg.	
			−Improvement in metabolic perturbations caused by obesity, both concentrations of extract.	
In vitro	Ethanolic Extract of Fruit and Leaves	0.2 mg/mL in vitro	−Inhibited LPL activity.	Pak-Dek et al. [50]
In vitro	Ethanolic Extract of Fruit	1 mg/mL in vitro	−Inhibited LPL activity.	Sahib et al. [51]

Effects of administration of different doses and parts of the *Morinda citrifolia* plant on obesity in in vivo and in vitro studies.

**Table 3:** The effects of administration of the *Morinda citrifolia* L. plant on insulin resistance/type 2 diabetes mellitus (T2DM).

Host	Part of Plant	Dose/Time	Effects	Reference
In vitro	*Morinda citrifolia* powder (plant part not identified) Methanol extract.	−Isolation of compounds (two new lignans, three new neolignans, and 10 known acid compounds).	−Stimulatory effects on glucose uptake through a fluorescent-tagged glucose probe (2-NBDG) in 3T3-L1 adipocyte cells.	Nguyen et al. [61]
			−Inhibitory effects on protein tyrosine phosphatase 1B gene (PTP1B), which is overexpressed in insulin resistance.	
Mice	Fruit (Fermented noni juice).	−1.5 µL/g body weight/twice daily/12 weeks	−Inhibited weight gain after 12 weeks.	Nerurkar et al. [49]
			−Improved glucose and insulin tolerance and fasting glucose in HFD-fed C57Bl/6 mice.	
			−Improved hepatic insulin resistance by FOXO-1 and inhibition of PEPCK and G6P (gluconeogenic enzymes)	
Mice	−Fruit (15 kg dried noni fruit powder fermented by Cheonggukjang and bacteria).	−fermented noni juice/90 days.	−Reduced fasting glucose levels, glycosylated hemoglobin (HbA1) in KK-Aγ diabetic-mice.	Lee et al. [76]
			−Improved insulin sensitivity in KK-Aγ diabetic-mice.	
In vitro	−Fruit (70% methanol extract).	−200 and 400 µg/mL in vitro.	−Diminished lipid fraction LDL and triglycerides in KK-Aγ diabetic-mice.	
			−70% methanol extract in culture cells (C2C12 cells) activated PPAR-γ and stimulated AMPK pathway.	
Mice	−Roots (methanol extract—soluble phases: CHCl_3_, EtOAc, *n-*BuOH, H_2_O).	−3 g/kg/single administration.	−*n*-BuOH fraction significantly reduced blood glucose levels after five hours of administration in streptozotocin-diabetic ddY mice.	Kamiya et al. [73]

Effects of administration of different doses and parts of the *Morinda citrifolia* plant on insulin resistance/T2DM in in vivo and in vitro studies.

**Table 4:** Effects of the administration of the *Morinda citrifolia* L. plant on non-alcoholic fatty liver disease (NAFLD).

Host	Part of Plant	Dose/Time	Effects	Reference
Hamster	Fruit noni juice (2.14 g crude polysaccharides/100 mL)	−3 mL (including 64.23 mg crude polysaccharides/kg body weight/6 weeks.	−Diminished visceral fat in HFD-hamsters (all doses).	Lin et al. [43]
			−Lowered serum and liver lipids: total cholesterol and triglycerides in HFD hamsters (all doses).	
		−6 mL (including 128.46 mg crude polysaccharides/kg body weight/6 weeks.	−Beneficial effects on the liver and hepatic enzymes (ALT) in HFD hamsters (all doses).	
			−Increased antioxidant capacity in the liver in HFD hamsters (all doses).	
		−9 mL (including 192.69 mg crude polysaccharides/kg body weight/6 weeks).	−Diminished inflammatory biomarkers in the liver (TNF-α, MCP-1, IL-1β) in HFD hamsters (all doses).	
			−Lowered gelatinolytic levels of MMP9 in HFD hamsters (all doses).	
Mice	Fruit fermented noni juice	−1.5 µL/g body weight/twice daily/12 weeks.	−Inhibited weight gain after 12 weeks.	Nerurkar et al. [49]
			−Improved glucose and insulin tolerance and fasting glucose in HFD-fed C57Bl/6 mice.	
			−Improved hepatic insulin resistance by FOXO-1 and inhibition of PEPCK and G6P (gluconeogenic enzymes).	

Effects of administration of different doses and parts of the *Morinda citrifolia* plant on NAFLD in in vivo studies.

**Table 5:** The effects of administration of the *Morinda citrifolia* L. plant on dyslipidemia.

Host	Part of Plant	Dose/Time	Effects	Reference
Rats	Fruit (Noni juice)	−50 mg/kg/day/30 days.	−Reduced body weight (better at 50 mg/kg/day dose).	Shoeb et al. [111]
		−100 mg/kg/day/30 days.	−Reduced serum total cholesterol, triglycerides, and lipids fractions: LDL and VLDL (all doses).	
			−Increased lipid fraction HDL (all doses).	
Rats Mice	Fruits, leaves and roots (70% ethanolic aqueous extract).	−Fruit ethanol extract (1000 and 500 mg/kg/day).	−All of the extracts on tyloxapol-induced hyperlipidemia: reduced total cholesterol and triglyceride levels.	Mandukhail et al. [154]
		−Leaf ethanol extract (1000 and 500 mg/kg/day).	−MFE in HFD-induced dyslipidemia (1000 mg/kg/day): prevented the rise of serum total cholesterol, LDL, total cholesterol/HDL ratio, and atherogenic index. No significant effects on HDL and glucose levels. No effect on body weight.	
		−Root ethanol extract (500 and 300 mg/kg/day)	−MLE in HFD-induced dyslipidemia (1000 mg/kg/day): prevented the rise in serum total cholesterol, LDL, total cholesterol/HDL ratio, atherogenic index, and glucose levels. No significant effects on HDL. Significantly prevented the gain in average body weight.	
			−MRE in HFD-induced dyslipidemia (500 mg/kg/day): prevented the rise in serum total cholesterol, LDL, total cholesterol/HDL ratio, atherogenic index, and glucose levels. Increased HDL. Significantly prevented the gain in average body weight.	
In vitro	Fruit (methanolic extract-soluble phases: CHCl_3_, EtOAc, nBuOH, H_2_O).	−Effective EtOAc purified and isolated lignans	−Lignans isolated from EtOAc fraction of methanol extract inhibited activity against copper-induced LDL oxidation by measuring the decrease in TBARS.	Kamiya et al. [60]
Hamster	Fruit (Fermented Noni Juice).	−3 mL NJ (containing 0.20 g solids/kg body weight)/day/6 weeks.	−Reduced sizes of heart, liver and visceral fat in HFD-cholesterol hamsters.	Lin et al. [48]
		−6 mL NJ (containing 0.40 g solids/kg body weight)/day/6 weeks	−Decreased serum triglycerides, total cholesterol, atherogenic index, malondialdehyde levels and hepatic lipids in HFD-cholesterol hamsters.	
		−9 mL NJ (containing 0.60 g solids/kg body weight) /day/6 weeks.	−Increased trolox equivalent antioxidant capacity (TEAC), glutathione (GSH), fecal lipids in HFD-cholesterol hamsters.	
			−Downregulated sterol regulator element binding protein-1c (SREBP-1c) and upregulated hepatic peroxisome proliferator-activated receptor-alpha (PPAR-α) and uncoupling protein 2 (UCP-2) mRNA in HFD-cholesterol hamsters.	

Effects of the administration of different doses and parts of the *Morinda citrifolia* plant on dyslipidemia in in vivo and in vitro studies.

**Table 6:** The effects of the *Morinda citrifolia* plant on hypertension.

Host	Part of Plant	Doses/Time	Effects	Reference
Rats	Fruit and leaf (ethanolic extract)	−Ethanolic extract of leaves (500 mg/kg body weight)/14 days	−Reduced blood pressure in dexamethasone-induced hypertensive rats (all doses and extracts).	Wigati et al. [55]
		−Ethanolic extract of fruit (500 mg/kg body weight)/14 days	−The highest hypotensive effect was with the combination of the extracts.	
		−Ethanolic extract of leaves + fruit (1:1, 500 mg/kg body weight)/14 days		
Rabbit, Rat and Guinea-pig	Roots (70% ethanolic- aqueous extract)	−Different concentrations on rabbit jejunum, thoracic aorta of rats, guinea pig atria in vitro * study.	−Rabbit jejunum: produced concentration-dependent relaxation of spontaneous and high K^+^-induced concentrations (antispasmodic effect).	Gilani et al. [93]
		−Positive control: verapamil (different concentrations in vitro * study).	−Guinea pig atria: caused inhibition of both atrial force and rate of spontaneous contractions (cardiopressant activity).	
		−* Concentration-response curve.	−Rabbit thoracic aorta: suppressed contractions induced by phenylephrine (1.0 µM) in normal –Ca^+2^ and Ca^+2^ - free Krebs solution- and by high K^+^, like the positive control (verapamil).	
			−Rat thoracic aorta: also caused relaxation of the phenylephrine (1.0 µM)-induced contractions (vasodilatory activity).	
Rats	Fruit (noni fruit juice)	−5 mg/kg (single administration after 24 h).	−Increased urine volume in a dose-dependent manner (10 mg/kg higher than 5 mg/kg)	Shenoy et al. [63]
		−10 mg/kg (single administration after 24 h).	−Decreased ion excretion (sodium and potassium)	
			−Aquaretic action.	

Effects of administration of different doses and parts of *Morinda citrifolia* plant on hypertension in in vivo studies. * Studies involving animal tissues in an in vitro assay.

**Table 7:** Effects of administration of *Morinda citrifolia* L. on gut microbiota.

Host	Methods	Effects	Reference
In vitro	Time courses of lactic acid fermentation of noni juice by *Lactobacillus casei*, *Bifidobacterium longum* and *Lactobacillus plantarum*	−All reached almost 10 × 10^8^ CFU/mL after 48 h of fermentation at 30 °C	Wang et al. [161]
In vitro	Human stool sample from a healthy volunteer (10 g) with ethanolic extracts of fermented noni fruit	−*Bifidobacterium* and *Lactobacillus* species, presented growth (0.16–0.63 mg/mL for *Bifidobacterium* and *Lactobacillus* spp.) compared with negative control (0 mg/mL)	Huang et al. [162]
Hybrid duck	Supplementation with noni fruit powder using different concentrations	−Increased amount of lactic acid bacteria with supplementation with 2% noni fruit powder;	Kurniawan, Widodo, Djunaidi [163]
		−Decreased *Escherichia coli* with supplementation with 3% noni fruit powder.	

Effects of administration of *Morinda citrifolia* on gut microbiota in in vivo and in vitro studies.
